# Enhancing generalizability theory with mixed‐effects models for heteroscedasticity in psychological measurement: A theoretical introduction with an application from EEG data

**DOI:** 10.1111/bmsp.70026

**Published:** 2026-01-18

**Authors:** Philippe Rast, Peter E. Clayson

**Affiliations:** ^1^ Department of Psychology University of California Davis Davis California USA; ^2^ Department of Psychology University of South Florida Tampa Florida USA

**Keywords:** generalizability theory, heteroscedasticity, mixed effects location scale model

## Abstract

Generalizability theory (G‐theory) defines a statistical framework for assessing measurement reliability by decomposing observed variance into meaningful components attributable to persons, facets, and error. Classic G‐theory assumes homoscedastic residual variances across measurement conditions, an assumption that is often violated in psychological and behavioural data. The main focus of this work is to extend G‐theory using a mixed‐effects location‐scale model (MELSM) that allows residual error variance to vary systematically across conditions and persons. By modeling heteroscedasticity, we can extend the computation of condition‐specific generalizability ($G_{t}$) and dependability ($D_{t}$) coefficients to reflect local reliability under varying degrees of measurement precision. As an illustration, we apply the model to empirical data from an EEG experiment and show that failing to account for variance heterogeneity can mask meaningful differences in measurement quality. A simulation‐based decision study further demonstrates how targeted increases in measurement density can improve reliability for low‐precision conditions or participants. The proposed framework retains the interpretative character of classical G‐theory while enhancing its flexibility. We argue that it supports finer‐grained insights on conditions that influence reliability and better‐informed design decisions in psychological measurements. We discuss implications for individualized reliability assessment, adaptive measurement strategies, and future extensions to multi‐facet designs.

## 1 INTRODUCTION

Behavioural measurements often involve multiple sources of variation, including person differences and various facets of the measurement process, such as variations stemming from different test items, different measurement occasions, different tasks, interactions thereof and unattributable measurement error. Classical Test Theory (CTT) is insensitive to most of these sources of variation and typically lumps all but the person differences (or other sources of true score variance) into the error variance (Lord & Novick, 1968; Spearman, 1910). While this is not necessarily a problem in itself, in many research scenarios this constraint can be limiting especially in the area of measurement reliability. As such, it is not surprising that Cronbach et al. (1963), whose works have been seminal in the area of psychological testing and measurement, proposed the Generalizability theory that yields a much more fine‐grained analysis and separation of potential sources of variation than CTT.

Generalizability theory (G‐theory) provides the framework for decomposing variance in behavioural measurements across multiple facets, such as tasks and raters, while treating persons as the object of measurement (Brennan, 2001). While G‐Theory was originally introduced to address shortcomings of CTT by decomposing error variability into different identifiable sources (facets) (Cronbach et al., 1963), its formal scope expanded with the introduction of the decision study (D‐study) framework to assess how reliability would change under alternative measurement designs (Cronbach et al., 1972). More recent applications have continued this expansion, including the evaluation of reliability across dynamic or complex conditions such as varying tasks, time points or populations (Vispoel et al., 2018).

To implement this framework in practice, G‐theory relies on a two‐part approach that includes a generalizability study (G‐study) to estimate variance components and a decision study (D‐study) to evaluate reliability under different measurement conditions. In a G‐study, variance components for each facet and their interactions are estimated using statistical models capable of separating sources of variation. Traditionally, analysis of variance (ANOVA) methods have been employed for this purpose. However, more recent approaches have incorporated structural equation modeling (SEM) and (multivariate) linear mixed‐effects models (Clayson & Miller, 2017a, 2017b; Jiang et al., 2020; Moore, 2016; Vispoel et al., 2023). The extracted variance components can then be used in a subsequent D‐study to predict the reliability of a measurement under various hypothetical design conditions (e.g., changing the number of items or sessions). By estimating how different facets contribute to total score variance, G‐theory extends CTT's single error term into a multifaceted error structure and provides not only a framework to quantify measurement error and reliability but also a principled approach to evaluating the impact on reliability due to different decisions regarding the study components.

A key assumption in the conventional application of G‐theory is that the residual (error) variance is homogeneous across all conditions or levels of facets (Brennan, 2010). In other words, the model traditionally assumes that measurement error has the *same distribution* regardless of the specific task, item, or occasion. In practice, however, this assumption of *homoscedasticity* may be violated. For example, in psychophysiological research, an electroencephalography (EEG) task with high cognitive load might produce more variable responses (larger within‐person error variance) than a simpler task, due to differences in attention or artifact rates (Clayson, 2024; Clayson & Miller, 2017b). Likewise, physiological measures such as heart rate can show much greater variability under stress conditions (e.g., during the Trier Social Stress Test Kirschbaum et al., 2008) than at rest. Ignoring heteroscedasticity, differences in residual variance across conditions or subpopulations, can lead to biased standard errors, misleading reliability estimates (Hayes & Cai, 2007; Jeanselme et al., 2025), and ultimately suboptimal decisions in study a design.

Indeed, the problem of heteroscedasticity in G‐theory has long been acknowledged (Brennan, 2001; Cronbach et al., 1972). Recent methodological developments have begun to explicitly model heteroscedasticity by relaxing the restrictive assumption of equal variance. For instance, Patz et al. (2002) introduced hierarchical rater models that allow rater‐specific severity and inconsistency but do not explicitly model scale parameters conditional on random effects. Generalized linear mixed models (Choi & Wilson, 2018) integrate G‐theory variance components into an IRT framework, relaxing normality and homoscedasticity assumptions but not explicitly introducing separate scale parameters. Bayesian nonparametric frameworks (Mignemi & Manolopoulou, 2024) handle heterogeneity through clustering and flexible distributions, yet they also do not explicitly model residual variance structures as scale parameters. Alternatively, practical methods like data splitting (Clayson, 2024) have been employed in situations where changes in residual variability over time are plausible, such as psychophysiological research involving heart rate variability (HRV). This approach divides continuous time series data into smaller segments or trials to separately estimate reliability, capturing variability obscured by treating the entire series as a single unit. By explicitly accounting for differences in measurement variability, these approaches improve reliability estimates; however, none have integrated specific scale models that handle heteroscedasticity within the classic G‐theory variance decomposition framework.

To address the limitations highlighted above and to provide a unified solution within the G‐theory framework, we propose explicitly incorporating mixed‐effects location‐scale models (MELSM; Hedeker et al., 2008; Rast et al., 2012). These models allow residual variances to differ systematically across various conditions (tasks, groups, items, etc.), enabling the direct modelling of heterogeneous residual variances. This approach removes the need for data splitting and allows systematic examination of temporal and contextual shifts in residual variability across both continuous and discrete facets within a single coherent analytic framework. However, while use of mixed effects location scale models in the estimation of condition‐, time‐ or individual‐specific reliability coefficients is not entirely new, it has not, to the best of our knowledge, been integrated into the classic G‐theory variance decomposition framework. Hedeker et al. (2008) introduced person and time‐dependent intraclass correlation coefficients (ICC) (see also Brunton‐Smith et al., 2017; Williams et al., 2021) and, more recently, Martinková et al. (2023) expanded this idea to the estimation of individual and covariate dependent inter‐rater reliability (IRR). While these methods all focus on reliability in the CTT setting, they share the same underlying statistical framework. Namely, these are scale models that allow one to explicitly incorporate the sources of heteroscedasticity and with it, allow one to estimate person and condition dependent reliability coefficients.

In this paper, we develop an extension of G‐theory integrating MELSMs to explicitly model heteroscedastic residual variances. We derive formulas for generalizability and dependability coefficients under this extended model. The next Section 2 reviews the necessary background and develops the proposed model, while Section 3 describes conducting D‐studies under heterogeneous residual variance conditions. Section 4 provides an illustrative application using a published psychophysiological. We close with a Section 5 on broader implications and practical recommendations for employing this heteroscedastic G‐theory framework.

## 2 TRADITIONAL GENERALIZABILITY THEORY

Consider an observed score $X_{pt}$ for person p on task (or item or condition) t in some measurement procedure. Classical test theory expresses this as $X_{pt}=T_{p}+E_{pt}$, where $T_{p}$ is the person's true score and $E_{pt}$ is an undifferentiated error term. G‐theory generalizes this by specifying multiple sources of variance. For example, in a one‐facet persons × tasks design, the observed score can be written as (Brennan, 2010):

(1) $$\begin{array}{llll} X_{pt} & =\mu \;\; & & \text{(grand mean)}\;\\ & \;+\nu_{p}\;\; & & \text{(person effect)}\;\\ & \;+\nu_{t}\;\; & & \text{(task effect)}\;\\ & \;+\nu_{pt}.\;\; & & \text{(residual)}\; \end{array}$$

Here, μ is the grand mean across all persons and tasks, where each ν term represents a random effect assumed to be normally distributed with mean zero and mutually independent. The residual term $\nu_{pt}$ captures the person‐by‐task interaction as well as any remaining variation not attributable to the main person or task effects. Under this model, the total variance of observed scores is decomposed as $\sigma_{X}^{2}=\sigma_{p}^{2}+\sigma_{t}^{2}+\sigma_{pt,e}^{2}$, where $\sigma_{p}^{2}$ is the universe‐score variance, $\sigma_{t}^{2}$ the variance due to different tasks, and $\sigma_{pt,e}^{2}$ is the residual variance.

In the traditional G‐theory framework, the *generalizability coefficient* G is defined as the proportion of variance attributable to universe scores relative to the variance that would cause persons to change rank ordering across replications. For the single‐facet design (person × task), the G‐coefficient is then defined as

(2) $$G=\frac{\sigma_{p}^{2}}{\sigma_{p}^{2}+\frac{\sigma_{pt,e}^{2}}{n_{t}^{\prime }}},$$

where $\sigma_{p}^{2}$ represents variance due to person differences, reflecting the universe score (or, in classical test theory terms, true score) variance. The term $\sigma_{pt,e}^{2}$ captures residual error, including the person‐by‐task interaction and any other unexplained variation. $n_{t}^{\prime }$ denotes the number of tasks. Note that the variance due to tasks themselves ($\sigma_{t}^{2}$) does not enter the denominator of the G‐coefficient because differences in task means do not affect the relative ordering of persons. Task variance is only relevant for absolute decisions, and therefore appears in the denominator of the D‐coefficient instead (discussed in Equation 3).

The generalizability coefficient in Equation (2) can be easily extended to a two‐facet design (person × task × occasion) that also accounts for measurement occasion, resulting in

$$G=\frac{\sigma_{p}^{2}}{\sigma_{p}^{2}+\frac{\sigma_{pt}^{2}}{n_{t}^{\prime }}+\frac{\sigma_{po}^{2}}{n_{o}^{\prime }}+\frac{\sigma_{pto,e}^{2}}{n_{t}^{\prime }n_{o}^{\prime }}}.$$

In this model, $\sigma_{pt}^{2}$ and $\sigma_{po}^{2}$ represent the person‐by‐task and person‐by‐occasion interaction variances, respectively, capturing how individual responses vary across tasks and occasions. The term $\sigma_{pto,e}^{2}$ is the residual variance, encompassing the three‐way interaction and other unmodelled noise. The $n_{o}^{\prime }$ denotes the number of occasions. The generalizability coefficient G thus reflects the proportion of variance due to persons relative to the total variance that contributes to inconsistency in rank‐ordering across tasks and replications. In classical G‐theory, this assumption of homoscedasticity can be made explicit by writing

$$X_{pt}=\mu +\nu_{p}+\nu_{t}+\nu_{pt},\;\nu_{p}∼𝒩(0,\sigma_{p}^{2}),\;\nu_{t}∼𝒩(0,\sigma_{t}^{2}),\;\nu_{pt}∼𝒩(0,\sigma_{pt,e}^{2}),$$

where $\sigma_{pt,e}^{2}$ is constant across all observations. This residual variance represents the combined person‐by‐task interaction and unsystematic measurement error. Importantly, the present extension does not alter this *location* structure. The mean model still follows the same random‐facet specification where persons and tasks remain random effects sampled from a universe of admissible observations. As such, the decomposition of the universe‐score and error components is preserved exactly as in traditional G‐theory. What changes is only the assumption on the residual variance: rather than assuming a single, constant $\sigma_{pt,e}^{2}$, we allow it to vary systematically across facets or covariates. In other words, the MELSM leaves the random‐effects (location) hierarchy intact while introducing a second‐level model that describes how the *scale* of the residuals may depend on observable features. Note that this standard model assumes *homoscedasticity*, that is, the residual variance $\sigma_{pto,e}^{2}$ is assumed to be constant across individuals. The primary aim of the present work is to relax this very assumption to allow $\sigma_{pto,e}^{2}$ to vary across persons, that is, to be *heteroscedastic*.

While the G‐coefficient is appropriate for norm‐referenced decisions, where rank ordering is the primary concern, the *dependability coefficient* (D) is used for absolute (criterion‐referenced) decisions, which are sensitive to both relative and absolute differences. The D‐coefficient for the same two‐facet design is defined as

(3) $$D=\frac{\sigma_{p}^{2}}{\sigma_{p}^{2}+\frac{\sigma_{pt}^{2}}{n_{t}^{\prime }}+\frac{\sigma_{po}^{2}}{n_{o}^{\prime }}+\frac{\sigma_{pto,e}^{2}}{n_{t}^{\prime }n_{o}^{\prime }}+\frac{\sigma_{t}^{2}}{n_{t}^{\prime }}+\frac{\sigma_{o}^{2}}{n_{o}^{\prime }}+\frac{\sigma_{to}^{2}}{n_{t}^{\prime }n_{o}^{\prime }}}.$$

Note that this formulation includes the additional terms $\sigma_{t}^{2}$, $\sigma_{o}^{2}$, and $\sigma_{to}^{2}$ that capture variance due to tasks, occasions, and their interaction. These components contribute to absolute error because they shift the overall level of observed scores across conditions. Given that the D coefficient is an extension of the G coefficient, the assumption of homoscedasticity remains in place.

However, if some facets are associated to systematically larger residual variances than others, the homogeneity assumption is violated. In such cases, a single $\sigma_{pto,e}^{2}$ may not adequately describe the data structure, and the reliability coefficients calculated from it may be misleading.

### 2.1 Modelling heterogeneous residual variances

To incorporate heterogeneous residual variances, we extend the G‐theory model to allow the variance of the residual term to depend on specific facets or conditions. Continuing with the simpler persons × task example, instead of a single $\sigma_{pt,e}^{2}$ for all tasks, we may allow the residual variance to differ across tasks:

(4) $$\mathrm{Var}(\nu_{pt}|t)=\sigma_{pt,e(t)}^{2}.$$

The term $\sigma_{pt,e(t)}^{2}$ denotes the residual variance for person p under condition t, with e(t) indexing the condition‐specific error variance. This formulation allows variance to vary smoothly across facets while being partially pooled across persons. The variance now conditions on t with the effect that the person‐by‐task interaction variance (or error variance) can be different for each task t. For example, Task 1 might have $\sigma_{pt,e(1)}^{2}=5$ (in whatever units the outcome is measured), while Task 2 has $\sigma_{pt,e(2)}^{2}=10$, indicating that Task 2 produces more erratic performance across persons (perhaps because it is ambiguously defined or especially difficult). Another way to interpret residual variance is in terms of consistency, where lower residual variance is associate with more consistent responses. Evidently, allowing for heteroscedasticity has a direct influence on the G‐theory coefficients that incorporate that conditional residual variance in (4), such as the G‐ and D‐coefficients.

More generally, in a multi‐facet design, we might find that the residual variance for the person‐by‐facet interaction differs by facet level. Consider a classic experimental design with persons p, tasks t, and multiple trials r per task. A typical G‐theory model could be expressed as

$$X_{ptr}=\mu +\nu_{p}+\nu_{t}+\nu_{pt}+\nu_{r(t)}+\nu_{pr(t)}+\nu_{ptr},$$

where μ is the grand mean, $\nu_{p}$ is the person effect, $\nu_{t}$ is the task effect, $\nu_{pt}$ is the person‐by‐task interaction, $\nu_{r(t)}$ is the trial effect nested within task, $\nu_{pr(t)}$ is the person‐by‐trial interaction nested within task, and $\nu_{ptr}$ is the residual error term. Note that, while this structure assumes trials are nested within tasks, alternative nesting schemes may be more appropriate in longitudinal or intensive repeated‐measures contexts. For example, when trials represent successive time points, it may be more natural to model tasks or conditions as nested within trials, allowing the variance structure to reflect temporal dynamics. This nesting reversal aligns with applications in experience sampling, daily diary studies or psychophysiological designs, where the primary source of variation may be time‐dependent rather than task‐dependent.

Additionally, one could generalize the model further by allowing other variance components, such as the person‐by‐trial interaction, to vary by facet level. For example, the variance of the person‐by‐trial interaction term may be modelled as $\mathrm{Var}(\nu_{pr(t)}|t)=\sigma_{pr,e(t)}^{2}$, allowing it to differ across tasks. This hierarchical modelling of variance heterogeneity goes beyond traditional G‐theory but can be useful when empirical evidence suggests structured differences in variability across conditions (see e.g., Brunton‐Smith et al., 2017, for a mixed effects location scale modeling approach that could be used to model between‐facet variances).

Conceptually, there are two approaches to modeling such heteroscedasticity. First, one may treat the differing conditions (e.g., tasks or items) as fixed facets and directly estimate separate variance component for each. This condition‐specific approach is akin to estimating error variances separately for each condition (e.g., $\sigma_{pi,e(1)}^{2},\sigma_{pi,e(2)}^{2},\text{…},\sigma_{pi,e(J)}^{2}$). A related strategy is the use of temporal data splits (Clayson, 2024), where time‐series data are divided into discrete segments (e.g., blocks of trials or epochs), and reliability is estimated separately within each. This approach does not model heteroscedasticity per se, but it does allow it to be detected and described.

Second, one may treat the facet as random and introduce a second‐level model for its variance. For instance, suppose tasks are randomly sampled from a universe of tasks, and their person × task interaction variance itself varies across tasks. We might model $\sigma_{pt,e(t)}^{2}$ as a random outcome from a distribution (e.g., log‐normal). This is essentially a *mixed‐effects location‐scale model* (MELSM) approach (Hedeker et al., 2008), where we have fixed and random effects for the means structure, or location, and fixed and random effects for residual variances, or scale part. In practice, this means adding a random effect that allows each task to have its own deviation in residual variance, or adding a random effect per person that allows each person to have their own residual scale, or both.

To make this concrete, consider the mixed‐effects location‐scale framework (Hedeker et al., 2008; Rast et al., 2012) with its notation adapted to mirror G‐theory standards. In this model, the usual mixed‐effects structure (5) is augmented with a submodel for the residual variance

(5) $$\begin{array}{ll} X_{pi} & =\mu +\nu_{p}+\nu_{i}+\nu_{pi},\; \end{array}$$

(6) $$\begin{array}{ll} \nu_{pi} & ∼𝒩(0,\sigma_{pi,e(i)}^{2}),\\ \sigma_{pi,e(i)}^{2} & =\exp\left(\alpha +\delta_{p}+\delta_{i}\right).\; \end{array}$$

Here, μ represents the fixed location intercept and the random effects $\nu_{p}$ and $\nu_{i}$ capture deviations from the mean due to person p and item i, respectively. The residual term $\nu_{pi}$ is normally distributed with mean zero and person–item‐specific variance $\sigma_{pi,e(i)}^{2}$. This variance is modelled via a log‐linear scale submodel defined in (6), where α is a fixed scale effect representing the overall log‐residual variance. $\delta_{p}$ and $\delta_{i}$ are random effects allowing the residual variance to vary across persons and items. This structure introduces heteroscedasticity directly into the residuals by modelling their residual variance as a function of random effects.

The MELSM further allows the facet specific random effects to correlate across location and scale, such that

(7) $$\begin{array}{ll} \zeta_{p}=\left[\begin{array}{c} \nu_{p}\\ \delta_{p} \end{array}\right]∼ & 𝒩\left(0,\Sigma_{\zeta }=\left[\begin{array}{cc} \sigma_{\nu ,p}^{2} & \\ \sigma_{\nu \delta ,p} & \sigma_{\delta ,p}^{2} \end{array}\right]\right),\; \end{array}$$

(8) $$\begin{array}{ll} \xi_{i}=\left[\begin{array}{c} \nu_{i}\\ \delta_{i} \end{array}\right]∼ & 𝒩\left(0,\Sigma_{\xi }=\left[\begin{array}{cc} \sigma_{\nu ,i}^{2} & \\ \sigma_{\nu \delta ,i} & \sigma_{\delta ,i}^{2} \end{array}\right]\right).\; \end{array}$$

Both, ζ and ξ contain the stacked location and scale random effects for person p and item i, respectively. These vectors are assumed to follow a bivariate normal distribution with mean zero and, in this case, a 2×2 covariance matrix Σ. Both of these covariance matrices contain the random effect variances in their diagonal and the covariance in the off‐diagonal element. For example, $\sigma_{\nu \delta ,i}$ captures the covariance between the location and scale random intercept of item i, linking deviations in mean response level to deviations in residual variability. Note that both the location and the scale submodels in (5) and (6) can include more random effects than merely a random intercept, increasing the dimension of the respective covariance matrices Σ beyond 2×2. In fact, in our Section 4 we will include random slopes for both submodels resulting in a 4×4 residual covariance matrix.

Note that the MELSM explicitly acknowledges heterogeneity in residual variances as well as mean‐variance dependence: both items and individuals may systematically differ in response consistency. The traditional homoscedastic model is recovered as a special case where $\delta_{i}=0$ and $\delta_{p}=0$ for all i,p. In that case, $\sigma_{pi,e(i)}^{2}$ reduces to $\sigma_{pi,e}^{2}=\exp(\alpha )$, and homoscedasticity becomes a testable assumption.

### 2.2 Reliability coefficients under heteroscedasticity

When residual variances differ across conditions (e.g., tasks or items), standard generalizability coefficients must be adapted to reflect this heterogeneity. One natural solution is to compute *condition‐specific reliability coefficients*.

#### 2.2.1 Condition Specific Generalizability Coefficient ${\text{G}}_{\text{t}}$

Suppose we have J fixed conditions t=1,…,J (e.g., specific tasks administered to all persons), each measured with $n_{t}^{\prime }$ replications (e.g., trials or raters). For each condition t, we define a condition‐specific generalizability coefficient as

$$G_{t}=\frac{\sigma_{p}^{2}}{\sigma_{p}^{2}+\frac{\sigma_{pt,e(t)}^{2}}{n_{t}^{\prime }}},$$

where $\sigma_{p}^{2}$ is the universe score variance (i.e., variance of person effects), and $\sigma_{pt,e(t)}^{2}$ is the residual variance associated with condition t. This residual variance may include a person‐by‐condition interaction and other condition‐specific error components, and it is allowed to vary across t. That is, each $G_{t}$ captures the expected reliability of measurements under condition t alone. For example, with two conditions t we can compute two generalizability coefficients $G_{1}=0.80$ for Task 1 and $G_{2}=0.60$ for Task 2, suggesting that Task 1 produces more consistent person‐level rankings than Task 2. Panel a in Figure 1 illustrates two tasks with different amounts for residual variance.

**FIGURE 1:**
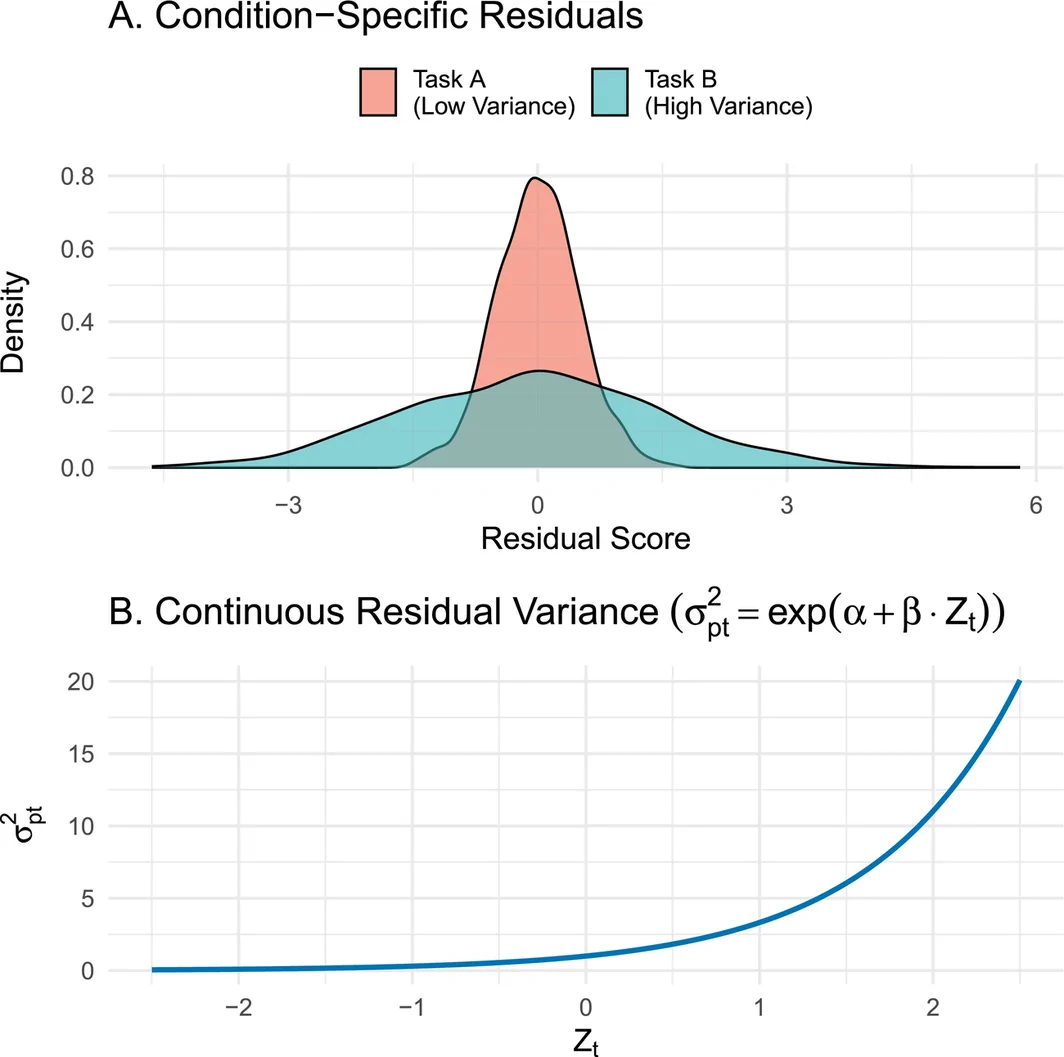
Illustration of heterogeneous residual variances. (Panel A) Distribution of residual scores for two tasks with different variances. Task A shows tighter clustering (lower variance) than Task B, indicating more consistent measurement. (Panel B) Residual variance $\sigma_{pt,e(t)}^{2}$ as a continuous function of a covariate $Z_{t}$, following Equation (9). This illustrates how heteroscedasticity can be modeled across a continuous predictor, rather than only across discrete conditions.

To move beyond condition‐specific estimates and enable generalizability coefficients that vary continuously (e.g., across elapsed time), we leverage the MELSM described above. The residual variance $\sigma_{pt,e(t)}^{2}$ is modelled using the scale submodel as defined in Equation (6) by further expanding it to include covariates so that

(9) $$\sigma_{pt,e(t)}^{2}=\exp\left(\alpha +\beta_{1}Z_{t1}+\beta_{2}Z_{t2}+\delta_{p}+\delta_{t}\right),$$

allowing $G_{t}$ to be modelled as a continuous function of predictors $Z_{1}$ and $Z_{2}$. To prevent further crowding of the residual variance term $\sigma_{pt,e(t)}^{2}$ we will keep this notation even if it contains continuous changes. The effect of the scale function with a continuous $Z_{t}$ on the residual variance under heteroscedasticity is illustrated in Panel b of Figure 1.

The expression in (9) defines a dynamic generalizability coefficient, where $G_{t}$ changes smoothly across conditions if $Z_{t}$ is continuous, or returns group‐specific reliability if $Z_{t}$ were to be dummy‐coded or factorized. This is a key contribution of our approach: instead of estimating isolated $G_{t}$ values, we obtain a general function that describes reliability as it varies across conditions – smoothly when modelled with continuous covariates, or discretely when modelled with factorized predictors. In doing so, the MELSM approach unifies condition‐specific and continuous reliability modelling within the generalizability framework.

#### 2.2.2 Condition‐specific dependability coefficient ${\text{D}}_{\text{t}}$

For absolute decisions where the concern is a person's observed score level on a specific condition t, we define a condition‐specific dependability coefficient as

(10) $$D_{t}=\frac{\sigma_{p}^{2}}{\sigma_{p}^{2}+\frac{\sigma_{pt,e(t)}^{2}}{n_{t}^{\prime }}+\frac{\sigma_{t}^{2}}{n_{t}^{\prime }}},$$

where $\sigma_{pt,e(t)}^{2}$ is the residual variance specific to condition t, and $\sigma_{t}^{2}$ is the variance due to condition means (i.e., how much conditions differ in their average score levels). This formulation assumes that each person completes $n_{t}^{\prime }$ replications of condition t, and that the condition (e.g., task) is treated as randomly sampled from a population. If the condition is treated as fixed, the $\sigma_{t}^{2}$ term may be omitted.

As with $G_{t}$, the dependability coefficient $D_{t}$ will be subjected to the same conditioning defined in Equation (9). As such, $D_{t}$ may be changing continuously or discretely across Z, depending on its scaling, and it may differ across facets $\delta_{p}$ and/or $\delta_{t}$:

$$D_{t}=\frac{\sigma_{p}^{2}}{\sigma_{p}^{2}+\frac{\exp\left(\alpha +\beta_{1}Z_{t1}+\beta_{2}Z_{t2}+\delta_{p}+\delta_{t}\right)}{n_{t}^{\prime }}+\frac{\sigma_{t}^{2}}{n_{t}^{\prime }}},$$

## 3 D‐STUDY WITH HETEROSCEDASTIC VARIANCE COMPONENTS

The purpose of a decision study (D‐study) was to use the variance components from a G‐study to project how measurement reliability would change under alternative designs. In standard G‐theory, residual variance is assumed to be homoscedastic, and adding more observations scales down error variance proportionally. In the heteroscedastic case, however, this logic no longer holds uniformly across conditions. That is, we can no longer assume that simply adding observations uniformly reduces error variance by a factor of n (the number of observations) for all conditions equally. Instead, the impact of adding observations may differ by condition, and the overall outcome depends on where those observations are added.

For example, consider a G‐study with J tasks, each measured with $n_{t}^{\prime }$ repeated trials. Suppose task‐specific residual variances ${\hat{\sigma }}_{pt,e(t)}^{2}$ and a universe score variance ${\hat{\sigma }}_{p}^{2}$ have been estimated. In the subsequent D‐study, we wish to determine the optimal allocation of trials across conditions t. Given the MELSM framework, the residual variance is predicted from the scale model (6)

$${\hat{\sigma }}_{pt,e(t)}^{2}=\exp\left(\hat{\alpha }+{\hat{\delta }}_{p}+{\hat{\delta }}_{t}\right),$$

and the effective residual variance when averaging $n_{t}^{\prime }$ trials is $\frac{{\hat{\sigma }}_{pt,e(t)}^{2}}{n{‘}_{t}}$.

Figure 2 illustrates both the continuous and conditional nature of $G_{t}$ for different effect sizes of Z, as well as the effect of different repeated trials per condition ($n_{t}^{\prime }$) and different amounts of universe scores $\sigma_{p}^{2}$. The effect of Z was weighted by illustrating four different scenarios ranging from no effect (β=0) to large positive changes (β=1.5) resulting small residual variances when *Z* is small and increasing variances as *Z* increases. Evidently, more repeated trials results in larger $G_{t}$ but the effect is does not play out linearly acorss Z ‐except when β=0. Larger universe scores (lower row) generally result in higher $G_{t}$ but again, due to the non‐linear effect of Z the gains or losses in reliability are not uniform.

**FIGURE 2:**
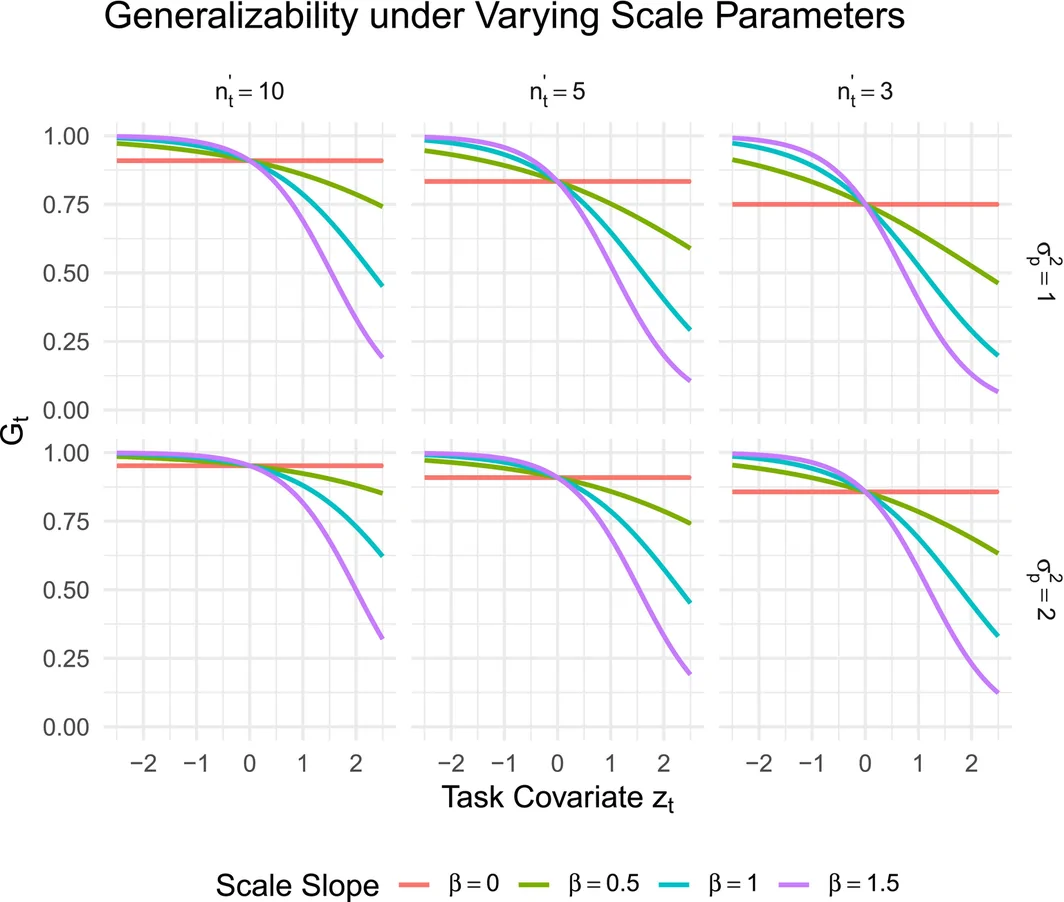
Projected generalizability coefficient $G_{t}$ as a function of a continuous task‐level covariate $z_{t}$ under varying assumptions about person‐level variance ($\sigma_{p}^{2}$), number of repeated trials per condition ($n_{t}^{\prime }$), and residual variance heterogeneity (scale slope β). Residual variances were modelled using a log‐linear scale model $\sigma_{pt,e(t)}^{2}=\exp(\alpha +\beta z_{t})$ with α=log(1.5). Each curve reflects a different value of β, controlling the degree of heteroscedasticity. Panels display how increases in $\sigma_{p}^{2}$ and $n_{t}^{\prime }$ improve generalizability, while steeper scale slopes β induce sharper drops in reliability for tasks with higher $z_{t}$. This illustrates how mixed‐effects location‐scale modelling enables dynamic, condition‐specific reliability estimation.

### 3.1 Estimation, parameters specification and model selection

#### 3.1.1 Model estimation

Heterogeneous variance models can be estimated using modern software. Maximum likelihood methods exist (e.g., using programs like MIXREGLS in Hedeker & Nordgren, 2013), although they are computationally more involved than standard linear mixed models and they tend to be limited in their ability to estimate larger MELSMs. A computationally more flexible alternative are Bayesian methods that provide a principled framework for estimating heteroscedastic G‐theory models. By assigning priors to variance components and sampling from the full posterior distribution, researchers can obtain condition‐ and person‐specific reliability estimates that naturally propagate uncertainty. Bayesian models also avoid issues such as negative variance estimates and perform well in small or unbalanced datasets (Klein et al., 2016; LoPilato et al., 2015).

These models can be estimated using Hamiltonian Monte Carlo (e.g., via Stan) or Gibbs sampling (e.g., via JAGS). In our application, we make use of the brms (Bürkner, 2017) package in R, which provides a convenient interface to Stan (Stan Development Team, 2016) and supports multilevel location‐scale modeling with correlated random effects. Posterior draws can be used to compute full distributions over generalizability coefficients $G_{t}$, as well as simulation‐based D‐studies. This makes the Bayesian framework particularly well‐suited for modern generalizability applications where heteroscedasticity and dynamic measurement structures are the norm.

#### 3.1.2 Prior specification

The priors adopted here are weakly informative and serve two purposes: (1) to stabilize estimation and constrain parameters to plausible ranges, and (2) to be sufficiently vague, allowing the data to dominate inference. While brms generally provides uninformative ‘flat’ default priors that meet these desiderata, we defined our own weakly informative priors for the present work because completely flat (improper uniform) priors are often unnecessarily uninformative and may yield unstable posteriors.

Given that the MELSM specifies separate submodels for the location (5) and the scale (6), priors were defined accordingly. For the fixed effects in the location submodel, we used zero‐centred priors that are mildly informative. Specifically, the prior distribution for the slope parameters in the location part of the model was defined as

$$\beta ∼𝒩\left(0,\sigma_{\mu }=10\right).$$

The prior for the intercept followed a zero‐centered Student‐t distribution to allow heavier tails, with ν=3 degrees of freedom and scale σ=5, allowing for potential large deviations:

$$\beta_{0}∼\text{Student‐}t\left(\mu =0,\nu =3,\sigma =5\right).$$

These priors are weakly informative, given that the data in our illustrative example were standardized. Random‐effect standard deviations followed half‐Student‐t distributions, defined as

$$\nu ∼\text{Student‐}t^{+}\left(\mu =0,\nu =3,\sigma =5\right),$$

where the “+” indicates truncation at zero.

For the fixed effects in the scale (α) submodel, we used a similar structure but with smaller scales. Note that the coefficients in the scale model are estimated on the log scale, which means the prior width must account for back‐transformation via exponentiation. The slope parameters were therefore defined as

$$\gamma ∼𝒩\left(0,\sigma_{\gamma }=1\right).$$

A $\sigma_{\gamma }=1$ prior corresponds to a 95% range on the original scale of approximately $e^{\pm 2}\approx [0.14,7.39]$, allowing the data to dominate the posterior. The intercept followed the same shape as for the location part but with a smaller scale:

$$\gamma_{0}∼\text{Student‐}t\left(\mu =0,\nu =3,\sigma =3\right).$$

Random‐effect standard deviations in the scale part followed half‐Student‐t priors with a tighter scale:

$$\nu_{\sigma }∼\text{Student‐}t^{+}\left(\mu =0,\nu =3,\sigma =2.5\right).$$

Correlations among random effects within the location and scale parts, both for persons (7) and items (8), were given independent LKJ priors with shape parameter η (Lewandowski et al., 2009). The random‐effect covariance matrices were decomposed as Σ=ΛΩΛ, where Ω is the correlation matrix of random effects and Λ is a diagonal matrix containing the random‐effect standard deviations defined above. Accordingly, the priors for the correlation matrices were specified as:

$$\Omega_{\nu }∼\text{LKJ}\left(\eta =1\right)\;\text{and}\;\Omega_{\delta }∼\text{LKJ}\left(\eta =1\right).$$

With η=1, the LKJ prior is uniform for a 2×2 correlation matrix. It is important to note that the LKJ prior is not scale‐invariant, and as the dimension of the correlation matrix increases, the marginal prior increasingly concentrates its mass around zero.

#### 3.1.3 Model selection

To evaluate whether modelling heteroscedasticity is warranted, we recommend comparing the heteroscedastic MELSM against a traditional homoscedastic model using predictive fit indices. Note that predictive accuracy measures do not require models to be nested, allowing comparisons between models that differ in their variance structure or covariate specification. In a Bayesian framework, predictive accuracy can be approximated using Pareto‐smoothed importance sampling leave‐one‐out cross‐validation (PSIS–LOO), which provides both the expected log pointwise predictive density (${\hat{\text{elpd}}}_{\text{loo}}$) for each model and the difference in their expected predictive accuracy (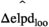; Vehtari et al., 2017). Following Sivula et al. (2022), comparisons are considered reliable when $|\Delta {\hat{\text{elpd}}}_{\text{loo}}|>4$; smaller values indicate that the models are too similar for the PSIS–LOO approximation to provide a stable ranking. To assess practical relevance, one can further examine the standard error of $\Delta {\hat{\text{elpd}}}_{\text{loo}}$, with differences exceeding twice the SE considered meaningful. Computing ${\hat{\text{elpd}}}_{\text{loo}}$ provides a Bayesian approach to assessing predictive fit and is asymptotically equivalent to the widely applicable information criterion (WAIC; Watanabe, 2010), which itself converges asymptotically to the Akaike information criterion (AIC; Akaike, 1998). Alternatively, models may be compared using marginal methods, such as the computation of Bayes Factors, which represent the odds in favour of a model with respect to competing models. Specifically, if models represent different competing hypotheses that come down to the comparison of a few model parameters, Bayes Factors might be fruitfully used (for an animated discussion on the contrast of these approaches see for example, Gronau & Wagenmakers, 2019; Navarro, 2019; Vehtari et al., 2019).

## 4 APPLICATION TO EMPIRICAL DATA

We illustrate the use of G‐theory with heterogeneous residual variance through an example in psychophysiological research, where multi‐facet designs and heteroscedastic measurement error are common. The principles generalize beyond neuroscience‐to educational testing, behavioural observation, ambulatory assessment and other settings where measurement error varies within or across individuals. Here, we demonstrate how trial‐level mixed‐effects location‐scale models (MELSM) can be used to estimate time‐varying generalizability and residual variability.

### 4.1 Clayson et al. Study on ERN dynamics

We reanalysed openly available data from Clayson et al. (2025), which measured trial‐level error‐related negativity (ERN) in a cognitive task battery. We focussed on the Flanker task and included *N* = 172 participants totalling 9810 observations. The outcome variable was single‐trial ERN amplitude, and the model allowed both the expected value and residual variance to vary as functions of time on task. The data and all accompanying documentation is available at https://osf.io/5rn6u, code to reproduce the statistical model as well as code to reproduce Figure 5 is provided in Figure B1. The data used in this study were collected in accordance with ethical standards, and informed consent was obtained by the original investigators. All participants provided written informed consent prior to study participation, which included information about data sharing and the related risks. Participants who did not consent to data sharing were not enrolled.

The model discussed here was estimated with (brms; Bürkner, 2017), a popular R software package that serves as wrapper for Stan (Stan Development Team, 2016). All predictors were standardized prior to model estimation, ensuring that the fixed‐effect priors operated on comparable scales across predictors. The model was fit with four chains, 2000 warm‐up and 2000 post‐warmup samples, totalling 8000 post‐warmup draws. To ensure good quality of the parameter estimates, we chose to keep the number of iterations at a level where the models converged with potential scale reduction factors $\hat{R}$ smaller than 1.1 (Gelman, 2006). All models were fit with the priors defined in the Section 3.1.2. The model converged on a standard desktop computer with multi‐threading (8 threads) in 70 s.

To distinguish within‐person dynamics from between‐person differences, we decomposed trial number into a person‐mean centred component (task time within‐person) and a subject‐level mean (task time between‐person). This separation, recommended in multilevel modelling (Curran & Bauer, 2011; Enders & Tofighi, 2007), prevents misattributing inter‐individual differences to intra‐individual change. We also included an ERN strength grouping variable (strong vs. weak), defined by a median split on participants' average ERN amplitude.

The model applied to the Flanker ERN data follows the mixed‐effects location‐scale structure and allows both the mean and residual variance to vary dynamically as a function of task time, group membership, and their interaction. Specifically, the model for the means structure was defined as:

$$\begin{array}{ll} \text{Location:}\; & {\text{ERN}}_{pt}∼𝒩(\mu_{pt},\sigma_{pt}^{2})\;\\ & \mu_{pt}=\beta_{0}+\beta_{1}{\left({\text{Time}}_{\text{within}}\right)}_{pt}+\beta_{2}{\left({\text{Time}}_{\text{between}}\right)}_{p}+\beta_{3}{\text{Group}}_{p}\;\\ & \;+\beta_{4}{\left({\text{Time}}_{\text{within}}\right)}_{pt}\times {\text{Group}}_{p}+\beta_{5}{\left({\text{Time}}_{\text{between}}\right)}_{p}\times {\text{Group}}_{p}\;\\ & \;+\nu_{0p}+\nu_{1p}{\left({\text{Time}}_{\text{within}}\right)}_{pt}.\; \end{array}$$

The submodel for the scale introduced the same predictors:

(11) $$\begin{array}{ll} \text{Scale:}\; & \log(\sigma_{pt})=\gamma_{0}+\gamma_{1}{\left({\text{Time}}_{\text{within}}\right)}_{pt}+\gamma_{2}{\left({\text{Time}}_{\text{between}}\right)}_{p}+\gamma_{3}{\text{Group}}_{p}\\ & \;+\gamma_{4}{\left({\text{Time}}_{\text{within}}\right)}_{pt}\times {\text{Group}}_{p}+\gamma_{5}{\left({\text{Time}}_{\text{between}}\right)}_{p}\times {\text{Group}}_{p}\\ & \;+\delta_{0p}+\delta_{1p}{\left({\text{Time}}_{\text{within}}\right)}_{pt}\; \end{array}$$

Note that in the location submodel, $\beta_{0}$ corresponds to the fixed intercept μ introduced in (5), and in the scale submodel, $\gamma_{0}$ corresponds to the fixed scale intercept α in (6). We adopt β and γ notation here to emphasize the inclusion of multiple covariates and interactions within each submodel.

The four person‐level random effects, $\nu_{0p},\nu_{1p}$ for the location submodel and $\delta_{0p},\delta_{1p}$ for the scale submodel, are jointly distributed as

$$\zeta_{p}={\left[\begin{array}{cccc} \nu_{0p} & \nu_{1p} & \delta_{0p} & \delta_{1p} \end{array}\right]}^{T}∼𝒩\left(0,\Sigma_{\zeta }=\left[\begin{array}{cccc} \sigma_{\nu_{0}}^{2} & & & \\ \sigma_{\nu_{1},\nu_{0}} & \sigma_{\nu_{1}}^{2} & & \\ \sigma_{\delta_{0},\nu_{0}} & \sigma_{\delta_{0},\nu_{1}} & \sigma_{\delta_{0}}^{2} & \\ \sigma_{\delta_{1},\nu_{0}} & \sigma_{\delta_{1},\nu_{1}} & \sigma_{\delta_{1},\delta_{0}} & \sigma_{\delta_{1}}^{2} \end{array}\right]\right),$$

where $\Sigma_{\zeta }$ is a 4×4 unstructured covariance matrix capturing not only variances within the location and scale models (captured in the diagonal of $\Sigma_{\zeta }$), but also covariances between the random intercepts and slopes across submodels (in the lower left (upper‐right) quadrant of the covariance matrix). For example, the covariance among the random location intercept and random scale intercept is captured in $\sigma_{\delta_{0},\nu_{0}}$.

This model differs from the simplified specification used earlier in the manuscript (see Equations 5 and 6), which included only random intercepts for each facet. By incorporating both random intercepts and random slopes for this illustration, the present model is meant to be closer to a real‐life application with a richer structure of individual differences. That is, here we allow participants to vary not only in their baseline ERN amplitude and trial‐level variability, but also in how these features change dynamically over time.

To evaluate whether accounting for heteroscedasticity improved model fit, we compared the heteroscedastic MELSM against a traditional homoscedastic mixed‐effects model. Model comparison using PSIS‐LOO showed a clear advantage for the heteroscedastic MELSM ($\Delta {\hat{\text{elpd}}}_{\text{loo}}=570.5$, SE =36.4), indicating substantially better predictive performance compared to the homoscedastic model. This difference exceeds both the reliability threshold ($|\Delta {\hat{\text{elpd}}}_{\text{loo}}|>4$) and the practical relevance criterion (difference >2 × SE; Sivula et al., 2022), supporting the inclusion of the scale submodel.

The results of the heteroscedastic model are reported in Table 1 and are briefly discussed here. Note however that the model in itself is not the focus of the paper and we chose to keep the discussion short. Overall, the results revealed several meaningful effects. In the location (mean) submodel, weak responders had significantly smaller negative ERNs overall ($M_{\text{ern\_groupweak}}=3.91$ with a 95% credible interval (CrI) [3.44, 4.39]). There was a modest positive within‐person effect of time ($M_{\text{task\_time\_within\_z}}=0.18$, 95% CrI [.00, .36]), indicating that ERN amplitudes became less negative (i.e., smaller in magnitude) as the task progressed (see also Clayson et al., 2025). This trend was attenuated or even reversed in weak responders, as indicated by a negative interaction ($M_{\text{task\_time\_within\_z}\times \text{group}}=-0.23$, 95% CrI [–.46, –.00]), suggesting that ERN amplitudes in the weak group remained stable or became slightly more negative over time. Note that the model uses treatment coding, with ‘strong’ responders as the reference group (coded as 0). The group coefficients reflect the deviation of ‘weak’ responders (coded as 1) from this reference.

**TABLE 1:** Fixed and random effects estimates from the MELSM for Flanker ERN.

Effect	Estimate	2.5%	97.5%	$\hat{R}$
Fixed effects (Location model)				
Intercept	−1.56	−1.92	−1.21	1.00
task_time_within_z	.18	.00	.36	1.00
task_time_between_z	−.03	−.34	.29	1.00
ern_groupweak	3.91	3.44	4.39	1.00
task_time_within_z × group	−.23	−.46	−.00	1.00
task_time_between_z × group	.35	−.16	.86	1.00
Fixed effects (Scale model)				
Intercept	1.59	1.53	1.66	1.00
task_time_within_z	.04	.01	.07	1.00
task_time_between_z	−.04	−.09	.02	1.00
ern_groupweak	−.05	−.13	.04	1.00
task_time_within_z × group	−.01	−.05	.03	1.00
task_time_between_z × group	.08	−.01	.17	1.00
Random effects				
SD (location intercept) $\nu_{0p}$	1.45	1.26	1.66	1.00
SD (location slope) $\nu_{1p}$	.31	.13	.48	1.00
SD (scale intercept) $\delta_{0p}$	.26	.23	.29	1.00
SD (scale slope) $\delta_{1p}$	.08	.06	.10	1.00

*Note*: Statistics of the posterior distribution of the fixed and random effects. Estimate reports the posterior mean, 2.5% and 97.5% are the lower and upper 95% Credible Intervals. $\hat{R}$ are the potential scale reduction factors. Random effects (SD) are standard deviations on their corresponding location and scale parameters. Estimates in the scale model are on the log scale. Notationally, in the interactions task_time_between_z × group, group is the short form of ern_groupweak. The reference group, coded as zero, are ‘strong’ responders while ‘weak’ responders are coded with 1.

In the scale submodel, residual variability increased reliably over time within individuals ($M_{\text{task\_time\_within\_z}}=0.04$, 95% CrI [.01, .07]), consistent with previous research (Clayson et al., 2025). The task_time_within_z × group interaction ($M_{\text{task\_time\_within\_z}\times \text{group}}=-0.01$, 95% CrI [‐.05, .03]) was basically zero indicating that the positive time trend held for both responder groups. Moreover, the “weak” responders were, on average, slightly less variable, but the difference was not meaningful. The positive between task_time_between_z × group interaction indicated that among participants who, on average, were located later in the task (i.e., had longer sessions), residual variability tended to be greater for weak responders (M=0.08). This suggests that longer or more extended testing sessions were associated with higher residual variance specifically in the weak group. While the 95% CrI [–.01, .17] of this interaction term included zero, 96.1% of the posterior samples were greater than zero, indicating that the probability of a positive effect is approximately p=.961.

The random effect SD's are reported in same Table 1 but are best visualized. Figure 3 shows the marginal of the posterior predicted residual SD's for each group over time inlcuding 95% credible interval bands, and Figure 4 reveals substantial heterogeneity in subject‐specific average SD trajectories. The individual trajectories are based on the posterior means from the scale model (11) of the fixed effects (γ's) plus the corresponding individual departures (δ's) encoded in the random effects. The random effects correlations were mostly zero and are reported in Table A1 in Appendix A.

**FIGURE 3:**
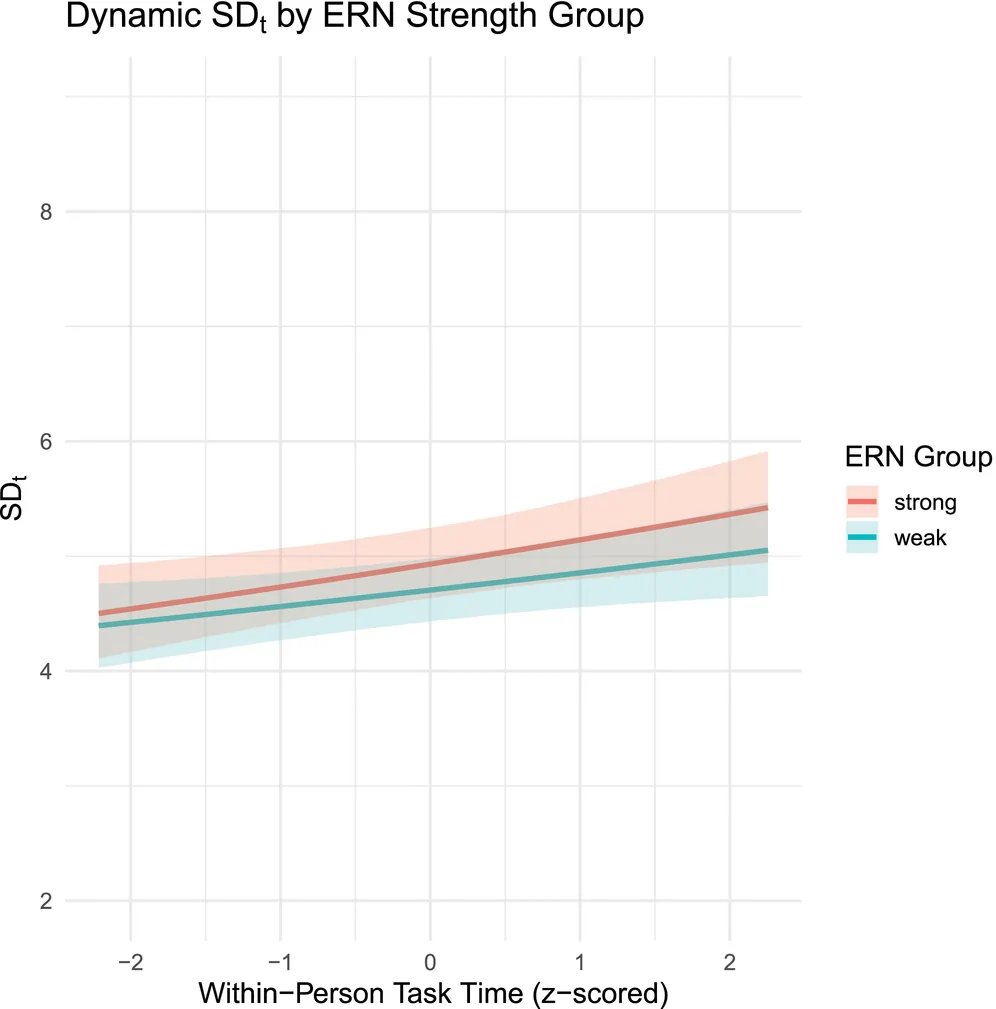
Estimated residual standard deviation ($\sigma_{t}$) over time for strong and weak ERN responders, marginalized across subjects. Shaded regions reflect 95% credible intervals.

**FIGURE 4:**
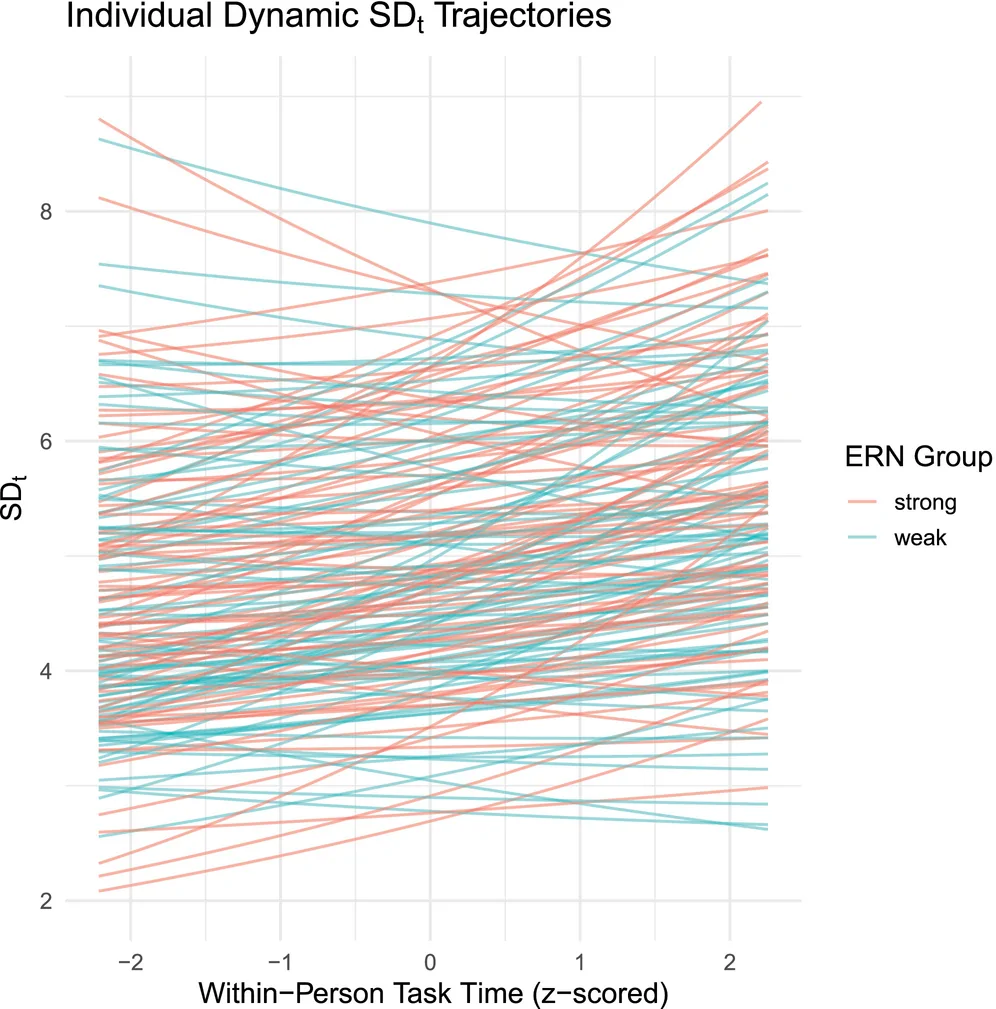
Subject‐specific residual standard deviation ($\sigma_{t}$) trajectories over time, coloured by ERN strength group. Each line represents one individual.

### 4.2 G‐study

The dynamic generalizability coefficient as was computed following Equation (2)

(12) $$G_{t}=\frac{\tau^{2}}{\tau^{2}+\frac{\sigma_{pt,e}^{2}}{n_{t}^{\prime }}},$$

where $\tau^{2}$ represents the between‐person variance, $\sigma_{pt,e}^{2}$ is the residual variance at time t for person p, and $n_{t}^{\prime }$ is the number of trials used to estimate a person's score at time t. In the present analysis, we set $n_{t}^{\prime }=1$ to reflect the generalizability of single‐trial scores over time. This reflects a G‐study perspective, where no aggregation across trials is assumed. The marginal estimates (Figure 5) showed that weak responders maintained slightly higher and more stable reliability over time. Individual trajectories (Figure 6) further revealed how reliability varied across persons and across trials. Both plots are based on the computed values that were used in the construction of Figures 3 and 4 but subsequently transformed according to Equation (12).

**FIGURE 5:**
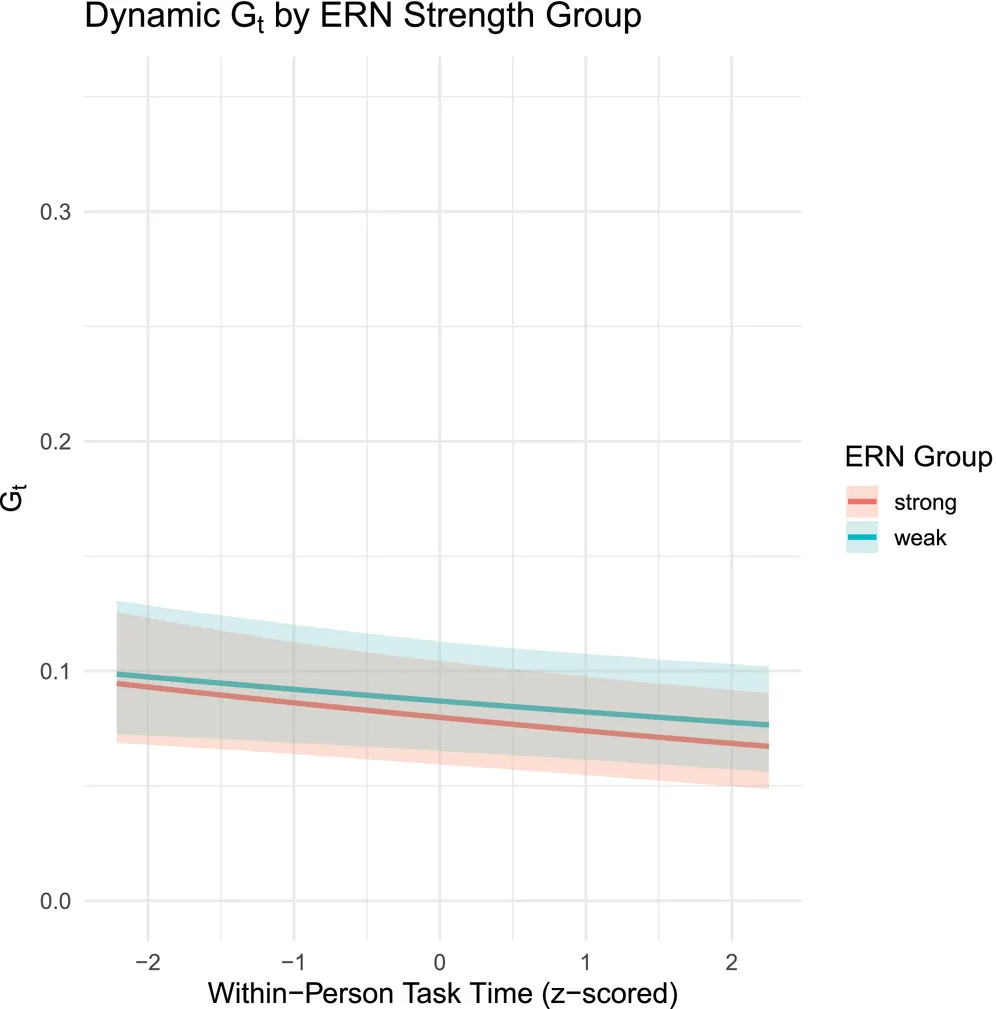
Dynamic generalizability coefficient ($G_{t}$) over time, computed according to Equation (12) from the posterior estimates of the between‐person variance ($\tau^{2}$) and the time‐specific residual variance ($\sigma_{pt,e}^{2}$). Both groups show a gradual decline in reliability across task time. Weak ERN responders exhibit slightly higher $G_{t}$ values on average, although credible intervals overlap, indicating modest group differences.

**FIGURE 6:**
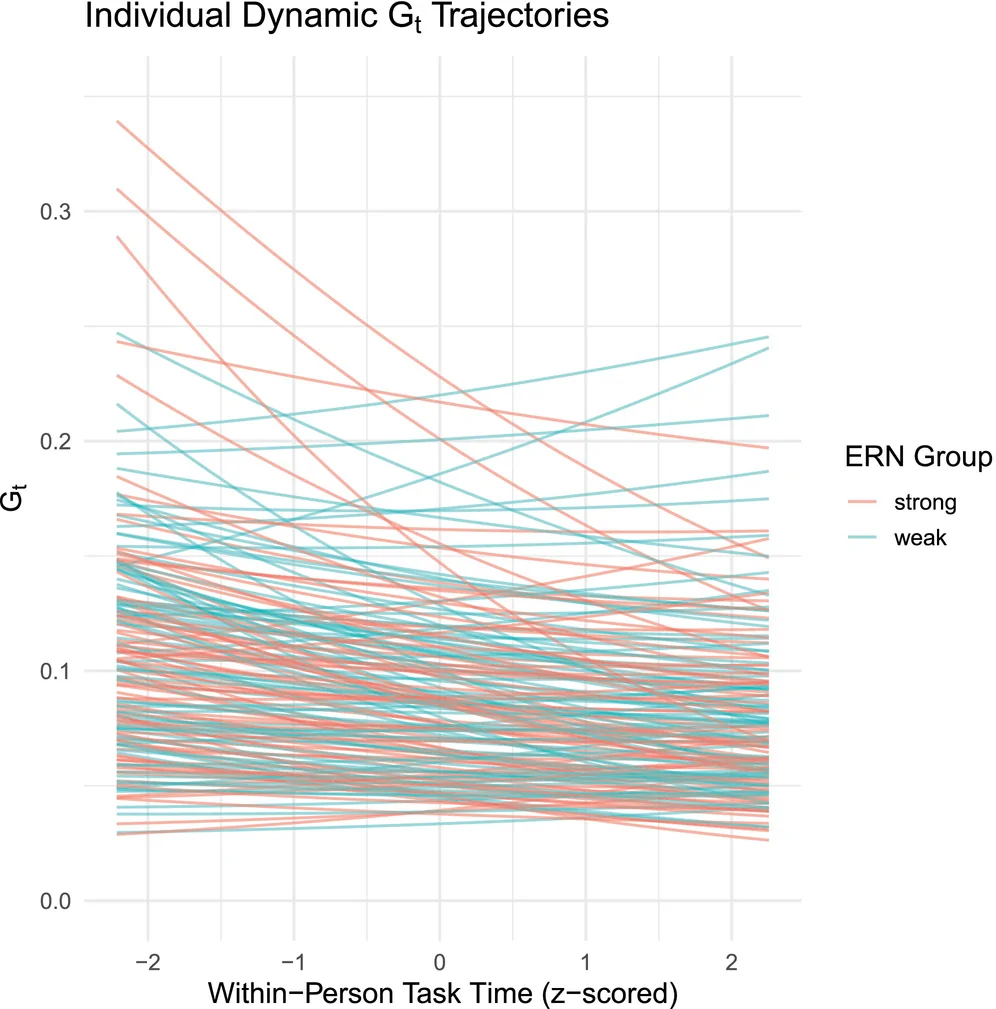
Individual‐level trajectories of the dynamic generalizability coefficient ($G_{t}$) across time. Note the substantial variability across participants, especially among weak ERN responders.

### 4.3 D‐study

Finally, we conducted a D‐study to simulate dependability ($D_{t}$) under increasing trial aggregation. Figure 7 illustrates changes in $D_{t}$ as a function of potential increases in trials averaged. As expected, $D_{t}$ substantially increased with more trials, reflecting improved reliability through aggregation. These differences between the aggregation levels are underscored by non‐overlapping 95% credible intervals (CrIs) indicating that dependability increases substantially with more trials. Given that weak responders tended to have slightly smaller residual variances, they also tended to have marginally larger dependability. The CrIs for both the weak and strong group, however, were largely overlapping indicating that these differences were negligible under different trial conditions and across different time points. These results show how dynamic G‐theory models can evaluate both measurement quality and design decisions, quantifying how reliability varies by group, person, and time.

**FIGURE 7:**
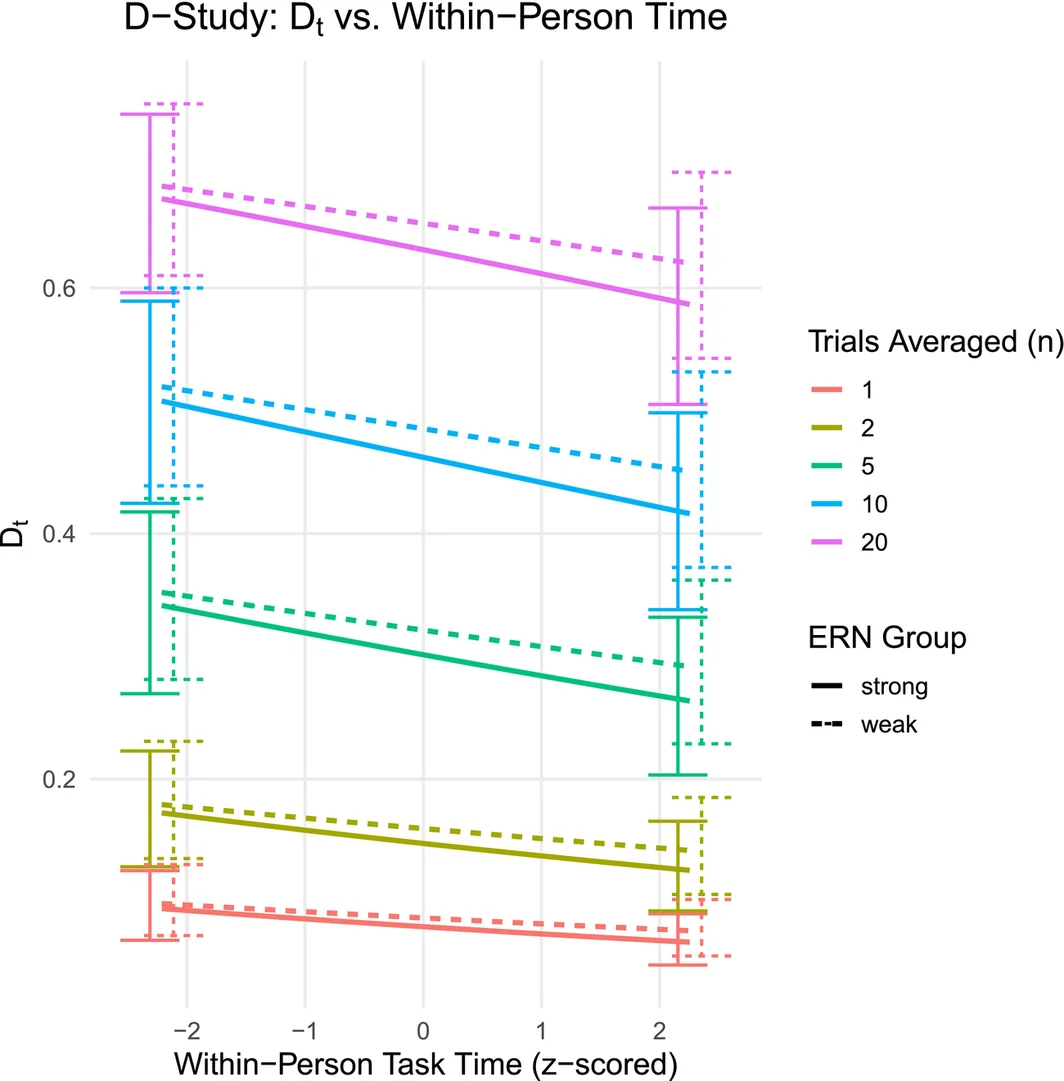
D‐study results showing how $D_{t}$ increases as a function of the number of trials averaged, for both ERN strength groups. Weak responders benefit substantially from aggregation, though a reliability gap remains.

To illustrate how D‐studies can inform design decisions, we can rewrite Equation (10) as a function of a desired dependability coefficient. Often D‐studies are conducted with respect to a fixed target criterion. For example, if we wish to determine the number of trials ($n_{t}^{\prime }$) required to reach the conventional target threshold of $D^{*}=.7$ (Cicchetti, 1994; Nunnally, 1978), we can solve Equation (10) for $n_{t}^{\prime }$:

$$n_{t}^{\prime }=\frac{\sigma_{pt,e(t)}^{2}+\sigma_{t}^{2}}{\sigma_{p}^{2}}\frac{D^{*}}{1-D^{*}}.$$

Here, $\sigma_{pt,e(t)}^{2}$, $\sigma_{t}^{2}$, and $\sigma_{p}^{2}$ are quantities estimated from posterior draws, allowing the uncertainty in these variance components to propagate directly to the resulting estimates of $n_{t}^{\prime }$. Figure 8 illustrates such a study with three target dependability levels ($D^{*}=0.6$, $D^{*}=0.7$, and $D^{*}=0.8$), representing cut‐offs that may be used in applied settings and are derived from intraclass correlation guidelines (Cicchetti, 1994).

**FIGURE 8:**
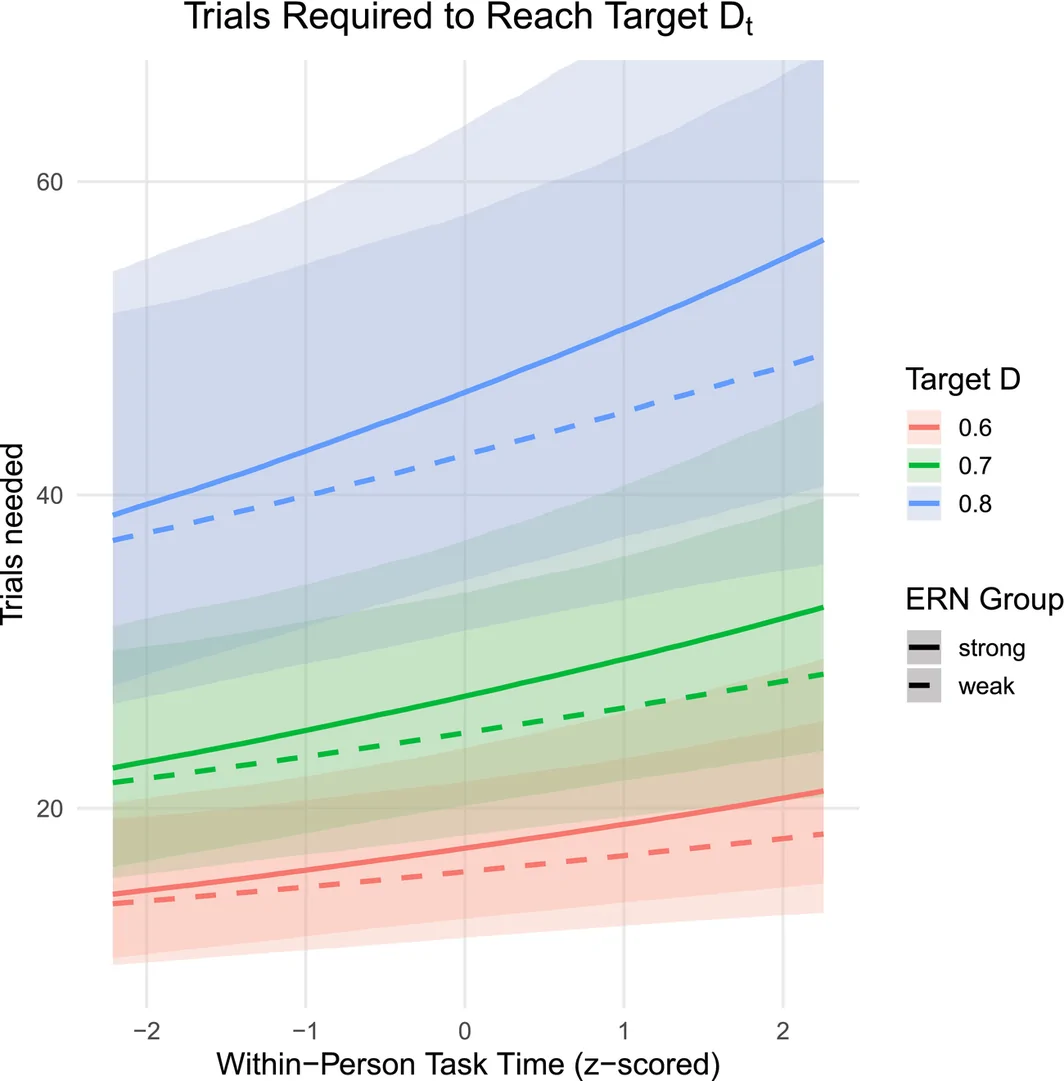
Number of trials required to reach target dependability ($D_{t}$) values of .6, .7 and .8 by within‐person task time and ERN group. Weak responders require slightly more trials to reach a given reliability threshold, but the overall pattern is similar across groups. An ideal target $D_{t}=.7$ is reached by the weak group with approximately 40 trials throughout the full time range. The bands represent 95% credible intervals.

### 4.4 Discussion of ERN dynamics results

Although reliability is typically defined at the group level, our dynamic G‐theory approach allows it to be estimated at much finer resolution, varying continuously over time and across individuals. Such subject‐ and time‐specific $G_{t}$ estimates are uncommon in standard applications of G‐theory, but they offer several diagnostic and theoretical advantages. For instance, they reveal not only average differences in reliability between groups (e.g., strong vs. weak ERN responders), but also which individuals are most stable at which points in the task. This insight could inform design decisions (e.g., knowing when reliability falls lets researchers trim tasks to their most reliable length or insert breaks where needed to reduced error variance) or suggest individual‐level exclusions (e.g., if a person exhibits unusually low reliability throughout), consistent with recent findings that reliability cutoffs can meaningfully alter sample composition and effect estimates (Heindorf et al., 2025).

Moreover, by modelling residual variance as a dynamic and potentially person‐specific quantity (via random slopes in the scale model), our framework naturally extends to the estimation of subject‐level error structure. Some individuals may be consistently more erratic than others but this type of information is lost in homoscedastic models. This opens doors to novel questions: for example, do clinical populations exhibit less stable measurement properties than controls, not only on average but in terms of residual consistency? Are some task conditions inherently more volatile than others? Does psychometric reliability of measurements vary systematically with psychiatric symptom severity? Such insights may inform both theoretical understanding and practical improvements in experimental design (see Clayson, 2024; Clayson et al., 2021, for applications in this realm) In this sense, heterogeneous residual modelling not only improves the accuracy of generalizability estimates but also enhances interpretability and adaptability, pointing to how and for whom a measure is stable. While we did not discuss a hypothesis‐based approach on whether certain design decision might meaningfully enhance a study, the Bayesian framework readily allows probabilistic evaluations of such substantive questions. Specifically, uncertainty in the G‐ and D‐study coefficients is fully propagated through the posterior distributions of all model parameters, so credible intervals, posterior probabilities, or ratios of evidence in favour (or against) group or time effects can be derived directly from these posteriors.

In the D‐study analysis, we explored how dynamic generalizability would change as a function of the number of trials averaged. While our main analyses treated each trial as a separate time point (i.e., $n_{t}^{\prime }=1$), the D‐study simulates scenarios where multiple trials are combined to estimate a person's score at each point in time. In practice, this might involve aggregating error trials within a block of trials (e.g., averaging all error‐related ERN amplitudes across a 1–2 min period), across a fixed number of adjacent trials (e.g., a moving window of 5 trials), or within meaningful task phases (e.g., early vs. late blocks). Each of these strategies increases the effective $n_{t}^{\prime }$, reducing residual error variance and improving reliability. By varying $n_{t}^{\prime }$ from 1 to 20, we model how reliability improves with additional observations, highlighting trade‐offs between temporal precision and measurement quality.

The empirical example illustrates several key advantages of modelling heteroscedastic residuals in generalizability theory. By relaxing the traditional assumption of equal error variances across conditions, our approach reveals substantial variation in reliability that would otherwise be obscured. For instance, in the EEG example, individuals with stronger responses exhibited lower generalizability, and task‐specific reliability estimates differed systematically across conditions. These findings are not merely technical; they have practical consequences for the design and interpretation of studies. Researchers working with psychophysiological data or behavioural measurements often face non‐uniform error structures, and our results suggest that overlooking this heterogeneity can lead to both over‐ and under‐estimation of reliability, depending on the condition. In applied settings, condition‐specific $G_{t}$ estimates can be used to guide data collection strategies, such as increasing the number of trials in lower‐reliability conditions or giving more weight to stable facets during composite score construction. Our D‐study simulations further show how measurement effort can be allocated efficiently: participants or conditions with initially low reliability benefit most from increased sampling, while others may require fewer repetitions. From a practical standpoint, the model supports diagnostic use cases, helping researchers identify poorly measured individuals or conditions that systematically compromise reliability.

## 5 GENERAL DISCUSSION

This paper set out to expand generalizability theory by integrating it with the mixed‐effects location‐scale modelling framework. Our aim was to show how structured heteroscedasticity, where residual variance varies across individuals, time points, or conditions, can be modelled explicitly within a generalizability‐theoretic approach. Using a trial‐level example from psychophysiological research, we demonstrated how reliability coefficients can be estimated dynamically, revealing time‐varying and person‐specific patterns of measurement precision. We also illustrated how these estimates can be extended to D‐study scenarios, guiding efficient design choices.

The ERN example highlighted several core features of this approach: It uncovered substantial variation in residual variability across groups and time compared to a standard mixed effects model with homogeneous residual variances, enabled estimation of individual reliability functions $G_{t}$, and revealed how trial aggregation affects dependability. These findings motivate a broader reconsideration of homoscedasticity assumptions in applied research, and point to the practical value of modelling variance structures in a principled and interpretable way.

In terms of broader implications, this work extends generalizability theory by embedding it within a mixed‐effects location‐scale model that allows for structured heteroscedasticity. Classical G‐theory has long acknowledged the possibility of unequal error variances, but in practice, nearly all applications assume homoscedasticity. The current integration preserves the random‐facet logic of classical G‐theory: persons, tasks, and other facets remain random effects in the location model, maintaining the universe‐score decomposition. The mixed‐effects location‐scale extension adds structure only to the residual variance, introducing fixed and random predictors of heteroscedasticity without altering the interpretation of the universe‐score variance or the random‐facet framework. Importantly, we retain the classical definitions of G and D coefficients, ensuring continuity with existing reliability metrics. What changes is the scope: rather than assuming a single pooled residual variance, we allow error to vary as a function of facets or covariates, leading to a generalizability function over conditions. This opens the door to more fine‐grained assessments of measurement quality and it aligns with psychology's shift towards more intensive longitudinal and idiographic designs (Hamaker, 2012; Molenaar, 2004).

Along these lines $G_{t}$ can provide person‐specific curves. While reliability has historically been defined at the level of the measurement procedure, our model estimates individual‐level trajectories that reflect differential error structure across people. The caveat here being that these curves should not be over‐interpreted as precise individual diagnostics as they are a function of estimated random effects from a population model. These random effects are subject to shrinkage and ergodicity (Molenaar, 2004); nonetheless, they might be useful tools for identifying participants whose measurements are consistently unreliable. In some cases, this may indicate disengagement or data quality issues; in others, it may suggest the need to adapt the measurement procedure to better suit certain individuals. As person‐centred and precision measurement approaches gain traction in psychology, tools that can characterize individual reliability will be increasingly valuable.

### 5.1 Limitations

At the same time, our approach is not without limitations. Modelling heteroscedasticity increases model complexity and demands more data to identify person‐ and condition‐specific variances. Bayesian estimation allows partial pooling and regularization, but researchers must still be mindful of the trade‐off between flexibility and overfitting. We recommend careful prior specification and convergence checks, especially when working with sparse or unbalanced designs. Moreover, while we focused on a single varying facet, the framework is readily extensible to multiple sources of residual heterogeneity, such as raters, items, or time. These extensions would increase computational burden but may prove useful in applications like social relations modeling or intensive longitudinal assessments.

Another aspect that we left unaccounted for is the possibility for heterogeneous random effects variances. These “structural” variances are sometimes modelled in the context of MELS models (Leckie et al., 2014; Rast & Ferrer, 2018), allowing an additional layer of variation in the random effects themselves. We chose not to include this level of modelling here because it substantially increases model dimensionality and parameter correlations, making estimation and interpretation considerably more complex. Also, the main focus was on residual variance heterogeneity that directly influences measurement precision and reliability within the G‐theory framework. We view such extensions as important directions for future work.

## 6 CONCLUSION

In summary, this paper offers a principled yet practical extension of generalizability theory that allows measurement precision to vary across tasks, persons, or time. It does so without abandoning the foundations of G‐theory, retaining its familiar variance decomposition and interpretive metrics. By bridging classical psychometrics and contemporary statistical modelling, our framework enables more accurate and context‐sensitive reliability estimates, providing both diagnostic insight and a path forward for designing more efficient, adaptive and fair measurement systems in psychological research.

## AUTHOR CONTRIBUTIONS

**Philippe Rast:** writing – review and editing; methodology; writing – original draft; conceptualization; formal analysis. **Peter E. Clayson:** resources; data curation; funding acquisition; investigation; writing – review and editing.

## CONFLICT OF INTEREST STATEMENT

The authors have declared no conflict of interest.

## DISCLOSURE OF ARTIFICIAL INTELLIGENCE‐GENERATED CONTENT (AIGC) TOOLS

In preparing this manuscript, ChatGPT‐4.0 was used to provide editorial assistance and to help adapt author‐generated R code into a minimal, reproducible example presented in Appendix B. All content, including intellectual contributions, analysis, and interpretation, originates from the authors. All AI‐assisted output was reviewed and corrected by the authors, who take full responsibility for the final manuscript.

## Figures and Tables

**FIGURE B1:**
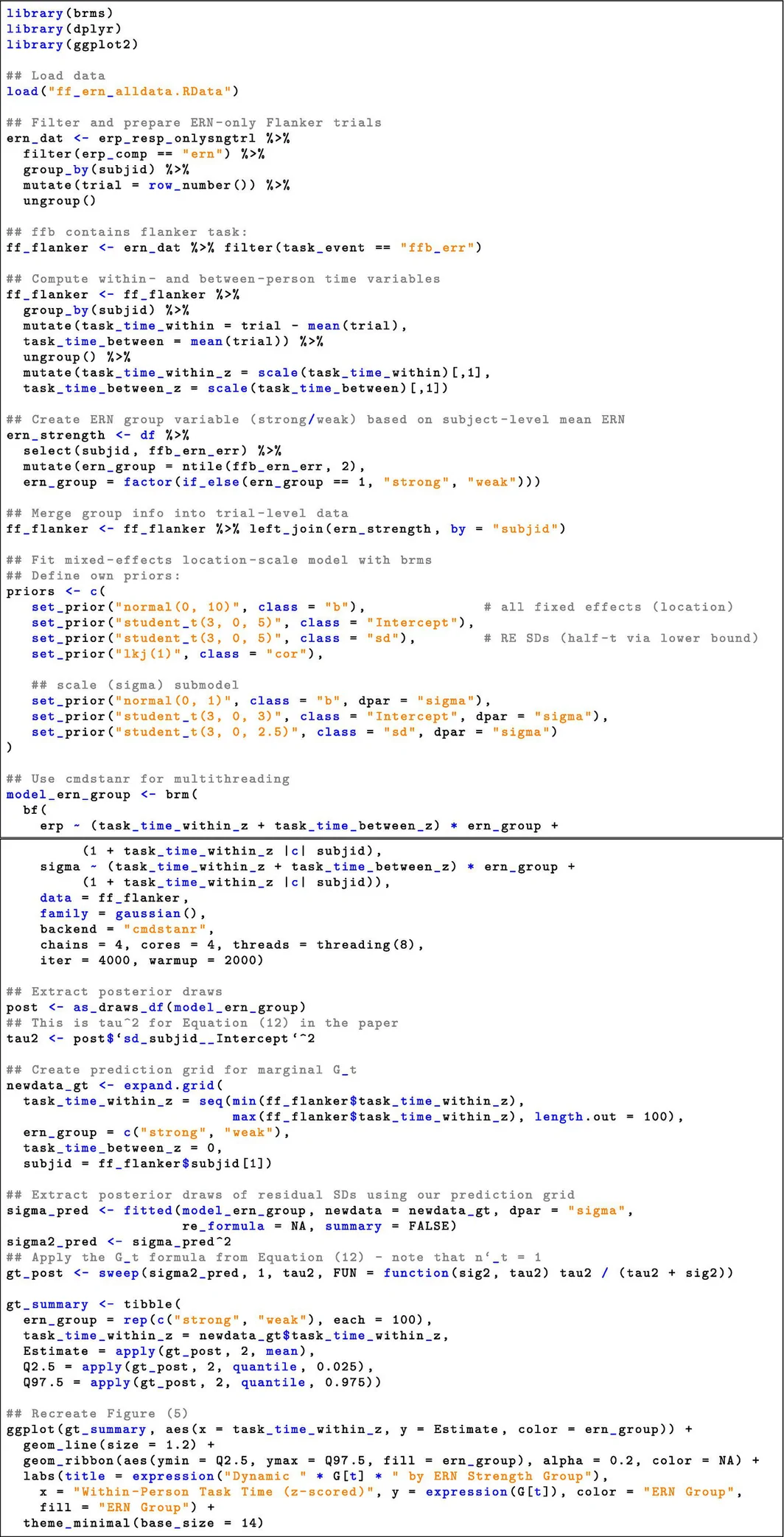
The RData file can be downloaded from the Github folder in the Files folder of the OSF project located at https://osf.io/5rn6u. AGI Note: ChatGPT 4.0 assisted in drafting the minimal, reproducible R code example, based on the original author generated code. The author (PR) reviewed, corrected, annotated, and tested this generated code.

## Data Availability

The data that support the findings of this study are openly available in osf at https://osf.io/5rn6u, reference number na.

## APPENDIX A LOCATION AND SCALE RANDOM EFFECT COVARIANCES

**TABLE A1:** Random effect correlations for location and scale submodels.

Parameter	Estimate	2.5%	97.5%	$\hat{R}$
$\text{Cor}(\nu_{0p},\nu_{1p})$	−.23	−.64	.16	1.00
$\text{Cor}(\nu_{0p},\delta_{0p})$	.03	−.15	.21	1.00
$\text{Cor}(\nu_{1p},\delta_{0p})$	.19	−.13	.57	1.02
$\text{Cor}(\nu_{0p},\delta_{1p})$	.15	−.11	.41	1.00
$\text{Cor}(\nu_{1p},\delta_{1p})$	−.11	−.63	.37	1.01
$\text{Cor}(\delta_{0p},\delta_{1p})$	−.07	−.32	.19	1.00

*Note*: Estimate report the posterior mean, 2.5% and 97.5% are the lower and upper 95% Credible Intervals. $\hat{R}$ are the potential scale reduction factors. Random effects (SD) are standard deviations on their corresponding location and scale paremeters. Cor's are the correlations among the random effects.
